# Use of high‐frequency ultrasound to study the prenatal development of cranial neural tube defects and hydrocephalus in *Gldc*‐deficient mice

**DOI:** 10.1002/pd.5004

**Published:** 2017-02-17

**Authors:** Maria C. Autuori, Yun J. Pai, Daniel J. Stuckey, Dawn Savery, Anna M. Marconi, Valentina Massa, Mark F. Lythgoe, Andrew J. Copp, Anna L. David, Nicholas D.E. Greene

**Affiliations:** ^1^ Newlife Birth Defects Research Centre and Developmental Biology and Cancer Programme, Great Ormond Street Institute of Child HealthUniversity College LondonLondonUK; ^2^ Department of Obstetrics and GynaecologySan Paolo HospitalMilanItaly; ^3^ Centre for Advanced Biomedical ImagingUniversity College LondonLondonUK; ^4^ Department of Health ScienceSan Paolo HospitalMilanItaly; ^5^ Maternal and Fetal Medicine, Institute for Women's HealthUniversity College LondonLondonUK

## Abstract

**Objective:**

We used non‐invasive high‐frequency ultrasound (HFUS) imaging to investigate embryonic brain development in a mouse model for neural tube defects (NTDs) and non‐ketotic hyperglycinemia (NKH).

**Method:**

Using HFUS, we imaged embryos carrying loss of function alleles of *Gldc* encoding glycine decarboxylase, a component of the glycine cleavage system in mitochondrial folate metabolism, which is known to be associated with cranial NTDs and NKH in humans. We serially examined the same litter during the second half of embryonic development and quantified cerebral structures. Genotype was confirmed using PCR. Histology was used to confirm ultrasound findings.

**Results:**

High‐frequency ultrasound allowed *in utero* detection of two major brain abnormalities in *Gldc*‐deficient mouse embryos, cranial NTDs (exencephaly) and ventriculomegaly (corresponding with the previous finding of post‐natal hydrocephalus). Serial ultrasound allowed individual embryos to be analysed at successive gestational time points. From embryonic day 16.5 to 18.5, the lateral ventricle volume reduced in wild‐type and heterozygous embryos but increased in homozygous *Gldc*‐deficient embryos.

**Conclusion:**

Exencephaly and ventriculomegaly were detectable by HFUS in homozygous *Gldc*‐deficient mouse embryos indicating this to be an effective tool to study CNS development. Longitudinal analysis of the same embryo allowed the prenatal onset and progression of ventricle enlargement in *Gldc*‐deficient mice to be determined. © 2017 The Authors. *Prenatal Diagnosis* published by John Wiley & Sons, Ltd.

## Abstract 2

**What's already known about this topic?**

- High‐frequency ultrasound (HFUS) has been used to non‐invasively monitor *in utero* mouse central nervous system (CNS) development during mid‐gestation (embryonic day 10.5–14.5); later gestational ages are yet to be studied. Missense mutations of the glycine decarboxylase gene *(Gldc)* are associated clinically with a metabolic disorder, Non‐ketotic hyperglycinemia (NKH) and neural tube defects (NTDs) such as exencephaly.

**What does this study add?**

- We extended HFUS imaging of the mouse CNS into late gestation embryonic day 18.5 in a genetic mouse mutant lacking *Gldc* in which NTDs, such as exencephaly, and hydrocephalus are prevalent. Serial HFUS can determine the age of onset of ventricle dilation that precedes hydrocephalus in this model.

## Introduction

The embryonic development of neural tube defects (NTDs) and other central nervous system (CNS) structural anomalies in humans is poorly understood. Studying animal models in which there are genetic mutations associated with abnormalities of CNS development are a useful method to determine timing of *in utero* pathology and possible therapeutic approaches.

High‐frequency ultrasound (HFUS) (30–100 MHz) allows non‐invasive visualisation of embryos within the uterus with a spatial resolution of <100 μm.^1^, ^2^, ^3^, ^4^ At the high frequencies used in developmental biology studies, soft tissue ultrasound penetration ranges from 5 to 10 mm,^3^ which is sufficient to image mouse embryos *in utero*. Being a non‐invasive technique, HFUS allows serial examination of the same litter during embryonic development, meaning that structural and functional embryonic development can be followed *in vivo* within the same embryo. Early studies using HFUS have visualised NTDs at embryonic day 10.5–11.5,^3^ and more recently, exencephaly, a cranial NTD that is the forerunner of anencephaly, and dilatation of the cerebral ventricles has been detected at E14.5 using this technique.^5^ A qualitative and quantitative evaluation of the volume of the ventricular system has been performed between E10.5 and 14.5.^1^ Later CNS development has not previously been studied using HFUS.

The glycine cleavage system (GCS) is a mitochondrial enzyme complex that participates in mitochondrial folate one‐carbon metabolism through decarboxylation of glycine and transfer of a one‐carbon group to tetrahydrofolate, generating 5,10‐methylene tetrahydrofolate.^6^ Missense mutations in *AMT* and *GLDC*, encoding components of the GCS have been identified in some patients affected by NTDs, including spina bifida and anencephaly, that result from incomplete closure of the neural tube.^7^, ^8^ Mutation of *GLDC* or *AMT* also cause non‐ketotic hyperglycinemia (NKH), a rare autosomal recessive disease, characterised by accumulation of glycine in body fluids and tissues,^9^ which typically presents in neonates, with lethargy, myoclonic jerks and respiratory problems, leading to coma and death.^10^, ^11^ In approximately 50% of NKH patients, there are additional structural brain abnormalities detected on MRI/CT scan during the newborn period such as agenesis and hypoplasia of the corpus callosum, abnormalities of the cerebellum and occasional hydrocephalus.^11^, ^12^, ^13^

Consistent with a role for *AMT* and *GLDC* in human NTDs and NKH, loss of function of the equivalent mouse genes, *Amt* and *Gldc*, causes a similar phenotype.^7^, ^14^ For example, in a *Gldc‐*deficient mouse strain, cranial NTDs occur in approximately 25% of homozygous mutants. The remaining homozygotes that are without NTDs show premature lethality with elevated levels of glycine in blood and urine,^14^ and approximately one third develop hydrocephalus. Histological analysis at E18.5 reveals enlarged lateral brain ventricles (ventriculomegaly) suggesting that the onset of hydrocephalus is prenatal.^14^ However, the pathogenesis and stage of onset of ventriculomegaly remain unclear. The lack of longitudinal data on brain development in *Gldc*‐deficient mice is a common feature of many animal models for genetic disease.

Using the ability of HFUS to provide longitudinal information in the same embryos, we aimed to characterise the ultrasound appearance of the developing mouse brain to follow the onset and progression of exencephaly (cranial NTDs) and ventriculomegaly, the two main abnormalities that are typical of *Gldc*‐deficient embryos. We extended the gestational age range of analysis to perform serial imaging of the developing brain in individual mouse embryos from the neurulation period to the end of pregnancy.

## Methods

### Animals

Animal studies were performed according to the UK Animals (Scientific Procedures) Act 1986 and the Medical Research Council's Responsibility in the Use of Animal for Medical Research (July 1993) at UCL Institute of Child Health and the Centre for Advanced Biomedical Imaging (CABI), University College London, London. Mice on a C57BL/6 background carried loss of function gene‐trap alleles of the glycine decarboxylase gene (here denoted respectively *Gldc* ^*GT1*^ and *Gldc* ^*GT2*^).^14^ *Gldc* ^*GT1/+*^ female mice were mated with *Gldc* ^*GT2/+*^ male mice overnight, and plugs were checked on the following morning. Embryonic day 0.5 (E0.5) of pregnancy was defined as morning of the day after overnight mating.

### Ultrasound procedures

Pregnant mice were transferred to the imaging centre and allowed to acclimatise for at least 48 hours before the first ultrasound scan. Ultrasound imaging was performed under general anaesthesia, induced with 4% isofluorane in oxygen (1 L/min) and maintained with isofluorane 2% in oxygen (1 L/min). Embryos were imaged using a Visualsonics Vevo 2100 imaging system (Visualsonics, Canada). The pregnant mouse was placed on the scan table (Visualsonics), with the limbs located on in‐built ECG electrodes to allow physiological monitoring. The fur was removed from the abdomen using a chemical hair remover, and pre‐warmed gel was applied as an ultrasound coupling medium. During the scan, body temperature was monitored using a rectal probe and maintained between 35 and 36 °C using an infrared lamp and heated stage. Respiratory and heart rates were monitored using external electrodes to monitor well‐being and to allow gating of the acquisition of 3D ultrasound volumes to respiration. Respiratory gating was used to avoid motion because of maternal respiratory movements, with a delay of 25% over a respiratory cycle after each breathing event, before data acquisition.

Scans were performed using two different microscan linear array transducers: Vevo 2100 MS550D (22–55 MHz) and MS700 (30–70 MHz) probes (Visualsonics). Maximum depth of penetration was 15 and 12 mm with a maximum image width of 14 and 9.7 mm respectively for the MS550D and MS700 probes. At the level of geometric focus, the axial and lateral resolutions were 40 and 90 μm for the MS550D probe and 30 and 75 μm for MS700 probe. A 3D motor stage connected both to the transducer and to the Vevo Imaging station allowed the acquisition of 3D volumes of the embryos with a step size of 32 μm. At the beginning of each ultrasound session, the maternal abdomen was scanned (MS550D probe); the maternal bladder, liver and spleen were identified and a 3 × 3 grid was drawn on the abdomen with a marker pen. Each embryo was distinguished in relation to its position in the uterine horns, its placental insertion and the maternal organs. Using the highest frequency probe (MS700 probe at E12.5 and E16.5, and the MS550D probe at E18.5), each identified embryo was then examined carefully in 2D for structural abnormalities: the brain was examined looking for any evident anomaly of the cerebral ventricles, of the cerebellum and the cortex. A 3D volume of the brain was then acquired and saved for later offline analysis. As far as possible, 3D volumes of the brain were obtained in the sagittal plane and in the transverse plane with midline structures equidistant to the skull, to optimise the identification of the ventricle diameters.

### Collection of embryos and histology

**Figure 1:**
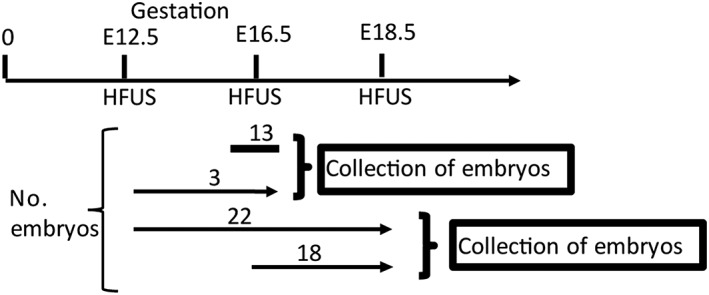
Experimental scheme for longitudinal assessment of brain development by high‐frequency ultrasound (HFUS): arrows indicate the number of embryos and gestational ages in days at which HFUS was performed for embryos that were analysed at multiple stages. Twenty‐two embryos were scanned at all three stages (E12.5, E16.5 and E18.5), 13 were scanned only at E16.5, and 18 were scanned at E16.5 and E18.5

After the last scheduled ultrasound scan (Figure 1), pregnant mice were sacrificed under general anaesthesia by cervical dislocation. The abdomen was opened; the uterus was removed and placed in Dulbecco's Modified Eagle's Medium (Invitrogen) containing 10% fetal calf serum. Each embryo was dissected from the uterus and examined for the presence of external abnormalities identified under ultrasound. Embryos were rinsed in phosphate‐buffered saline and fixed in Bouin's solution (after removal of the skin for litters at E18.5). For histological examination, embryos were dehydrated, embedded in paraffin wax, sectioned at 10‐μm thickness and stained with haematoxylin and eosin. Histological examination of individual embryos was performed blind to ultrasound qualitative and quantitative data.

### Genotyping of experimental litters

Embryos were genotyped by PCR amplification of genomic DNA, isolated from tail tip or limb, as described previously.^14^ Intron‐specific primers amplify products from wild‐type and heterozygous samples. Use of an intron‐specific primer with a gene‐trap construct‐specific primer generated 602‐bp and 325‐bp products corresponding to the *Gldc* ^*GT1*^ and *Gldc* ^*GT2*^ alleles, respectively.

### Manual segmentation and quantitative analysis of cerebral structures in three‐dimensional HFUS images

**Figure 2:**
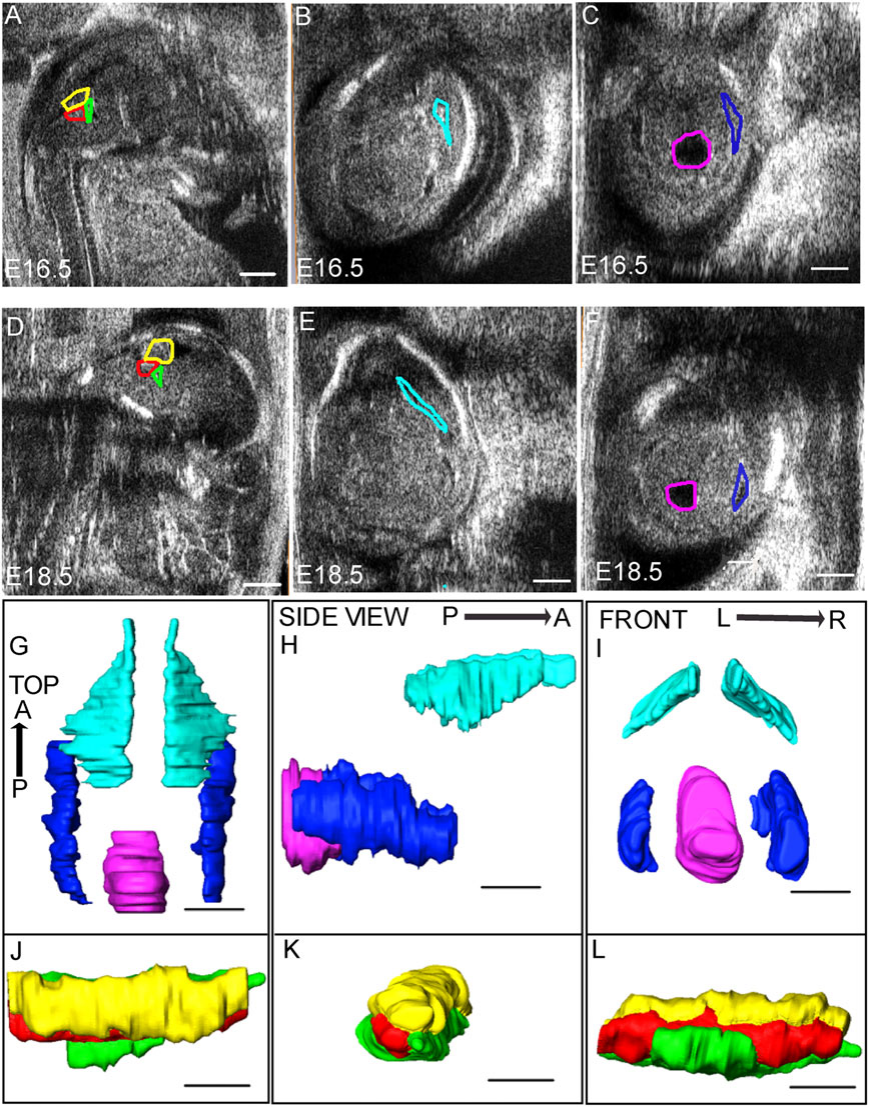
Manual segmentation of high‐frequency ultrasound images: images at E16.5 (A–C) and E18.5 (D–F) illustrate the regions defined for segmentation. The cerebellum, the fourth ventricle and its choroid plexus (yellow, green and red line, respectively) are measured in the sagittal plane (A and D). Images in the transverse plane (B, C, E and F) were used to outline the body of the lateral ventricles (light blue lines; B and E), the base of the posterior horn of the lateral ventricles (blue lines; C and F) and the mesencephalic vesicle (purple line; C and F). G–L show three‐dimensional images of cerebral structures after segmentation at E16.5. G‐I show surface rendering of the body and the base of the posterior horn of the lateral ventricles (light blue and blue, respectively) and the mesencephalic vesicle (purple). J–L show the surface rendering of the cerebellum (yellow), the fourth ventricle (green) and its choroid plexus (red). Anatomic axes for top view (G and J), side view (H and K) and front view (I and L) are indicated as anterior–posterior (A–P) and right–left (R–L). Scale bar represents 1 mm in all images

Offline examination of 3D volumes of brain structures was performed blind to genotype, using Vevo 2100 software (Visualsonics) and/or Amira software (FEI). A qualitative assessment was followed by manual segmentation of the structures of interest: mesencephalic vesicle, body of the lateral ventricles and base of the posterior horn of the lateral ventricles, fourth ventricle and its choroid plexus and cerebellum (Figure 2). The cerebral ventricles were recognised as anechoic fluid‐filled cavities surrounded by brain tissue. The lateral ventricles were divided into two parts: body of lateral ventricles and base of the posterior horn according to established anatomical criteria.^15^ Although these structures are connected, the base of the posterior horn and the body of the lateral ventricles were measured separately as the spatial resolution of HFUS was not sufficient to allow us to distinguish their point of connection distinct from the surrounding tissues. Ventricle volumes were normalised to the hemisphere size on the same side of the embryo, with laterality being identifiable on the basis of the position of the embryo in the whole 3D image. In transverse and coronal HFUS sections, the body of the lateral ventricles was measured as the part of the lateral ventricles just ventral and dorsal to the third ventricle. The base of the posterior horn of the lateral ventricles was recognised at its caudal part, at the level of the mesencephalic vesicle and fourth ventricle. The mesencephalic vesicle, precursor of aqueduct of Silvius, was recognised as a rounded shape fluid‐filled structure in the posterior part of the brain. The cerebellum was recognised as an echogenic structure, localized between the caudal part of the mesencephalic vesicle and the roof of the fourth ventricle. The fourth ventricle with its choroid plexus was identified as the most caudal part of the cerebral ventricular system.

The body of the lateral ventricles, base of the posterior horn of the lateral ventricles and the mesencephalic vesicle were measured in the transverse section (Figure 2), while the cerebellum and the fourth ventricle with its choroid plexus were measured in the sagittal section (Figure 2). When one or both of these sections were not achievable, structures of interest were measured in the coronal section (Figure 4). Manual segmentation was obtained by drawing the borders corresponding to signal intensity transition at the brain‐cerebrospinal fluid interface, with a step size of 0.1 mm. After segmentation, volume measurements were performed automatically using Vevo 2100 Workstation Software. Figure 2 shows the volume rendering of the cerebral structures in the three orthogonal planes. To account for potential variability in brain size owing to differing developmental stages of litters of the same gestational age (depending on time of mating), each absolute volume was normalised with respect to the cerebral hemisphere diameter. For consistency, the hemisphere diameter was measured in the same transverse section as were visualized the mesencephalic vesicle and the lateral and third ventricles, from the midline to the outer edge of the parietal bone.

Extraction of morphometric data using manual segmentation may vary by the same operator under different circumstances,^16^, ^17^ and we, therefore, assessed the intra‐observer variability. This part of the study was conducted using Amira software (FEI), which is not specific for the Vevo 2100 instrument.

### Statistical analysis

Each volume measurement was analysed as an absolute measurement and after normalisation to the cerebral hemisphere diameter. One‐way ANOVA was used for the analysis of measured parameters in comparison of multiple genotypes (*Gldc* ^*+/+*^, *Gldc* ^*GT1/+*^, *Gldc* ^*GT2/+*^ and *Gldc* ^*GT1/GT2*^) at E16.5 and E18.5, with post hoc analysis by Holm–Sidak test. Pairwise comparisons were made by *t*‐test where only two genotypes were compared. The obtained proportion of embryos for each genotype was compared with the expected Mendelian Ratio using Chi‐square test. Differences were considered significant when *p* < 0.05.

## Results

### Accuracy of qualitative and quantitative evaluation of cerebral structures

Scans were performed at three gestational ages as follows: E12.5 by which time primary and secondary neurulation have been completed, E16.5 to study the development of the cerebellum and the cerebral ventricles and E18.5, to investigate enlargement of the cerebral ventricles in the *Gldc*‐deficient mouse model. A total of 56 embryos from nine litters were imaged by HFUS and collected at the end of the experimental protocol. Of these, 40 embryos were imaged at two or more stages of pregnancy with the final scan at E18.5 (Figure 1). The time required for the acquisition of HFUS of the embryos from each litter was approximately 40 min. There were no experimental complications or embryonic losses, confirming that repeated HFUS during pregnancy is feasible in mouse models.

To quantify intra‐observer variability segmentation, analyses were carried out offline on the cerebral structures of interest of five embryos 1 week apart and with the second analysis carried out blind to sample identity and in a random order. For volumes of the various structures determined at E18.5, the mean coefficient of variation varied from 5.9 to 10.3%, with the exception of the cerebellum (18.6%) and fourth ventricle (21%). At E16.5, the mean coefficient of variation for measured volumes varied from 2.9 to 14.9%, with the exception of the mesencephalic vesicle (22.8%) and choroid plexus (23.0%).

The embryonic stage and size of the gestational sac affected the extent to which a morphological examination of the entire litter was possible. At E12.5, a morphological examination of all embryos was possible in 100% of pregnant dams. As gestation advanced and the embryonic dimensions increased, the evaluation of morphology became more challenging, because of the closer positioning of the amniotic sacs in the maternal abdomen or because of embryonic lie (cephalic, breech or transverse). A morphometric evaluation of the brain structures was achieved in 92% and 88% of embryos without NTDs at E16.5 and E18.5, respectively (Table 1). Exencephaly occurred in eight embryos and was detected by ultrasound on every occasion (16 out of 16 scans), regardless of the stage of gestation (Table 1)

**Table 1:** *In utero* ultrasound during mouse development

Embryonic day	E12.5		E16.5		E18.5	
	*n*	No. informative scans (%)	*n*	No. informative scans (%)	*n*	No. informative scans (%)
Total embryos	25	25 (100)	56	52 (93)	40	36 (90)
Embryos with NTDs	2	2 (100)	8	8 (100)	6	6 (100)
Embryos without NTDs	23	23 (100)	48	44 (92)	34	30 (88)

The total number of embryos scanned at each developmental stage is indicated, together with the number and proportion of embryos per litter for which informative scan images could be acquired.

NTD, neural tube defect.

All embryos were genotyped at the end of the experiments. There was no significant difference between the observed distribution of embryos among the four different genotypes and the expected Mendelian ratio (*p* > 0.05, χ^2^ test), suggesting the absence of early *in utero* lethality (Table 2). There were no embryos affected by exencephaly among wild‐type or heterozygous embryos, while exencephaly occurred among 44.4% of compound heterozygous embryos (effectively homozygous for a *Gldc* mutation), confirming that loss of *Gldc* expression causes cranial NTDs (*p* < 0.05) (Table 2).

**Table 2:** Frequency of genotypes and occurrence of NTDs among offspring of *Gldc* heterozygous matings

Genotype	*Gldc* ^*+/+*^ (%)	*Gldc* ^*GT1/+*^ (%)	*Gldc* ^*GT2/+*^ (%)	*Gldc* ^*GT1/GT2*^ (%)
No. collected (%)	16 (28.6)	15/56 (26.8)	7/56 (12.5)	18/56 (32.1)
No. expected (%)	14 (25)	14/56 (25)	14/56 (25)	14/56 (25)
No. NTDs (%)	0 (0)	0/15 (0)	0/7 (0)	8/18 (44.4)

The observed ratio of genotypes did not significantly differ from the predicted Mendelian ratio (*p* > 0.05).

### Ultrasound appearance of developing cerebral structures and exencephaly

**Figure 3:**
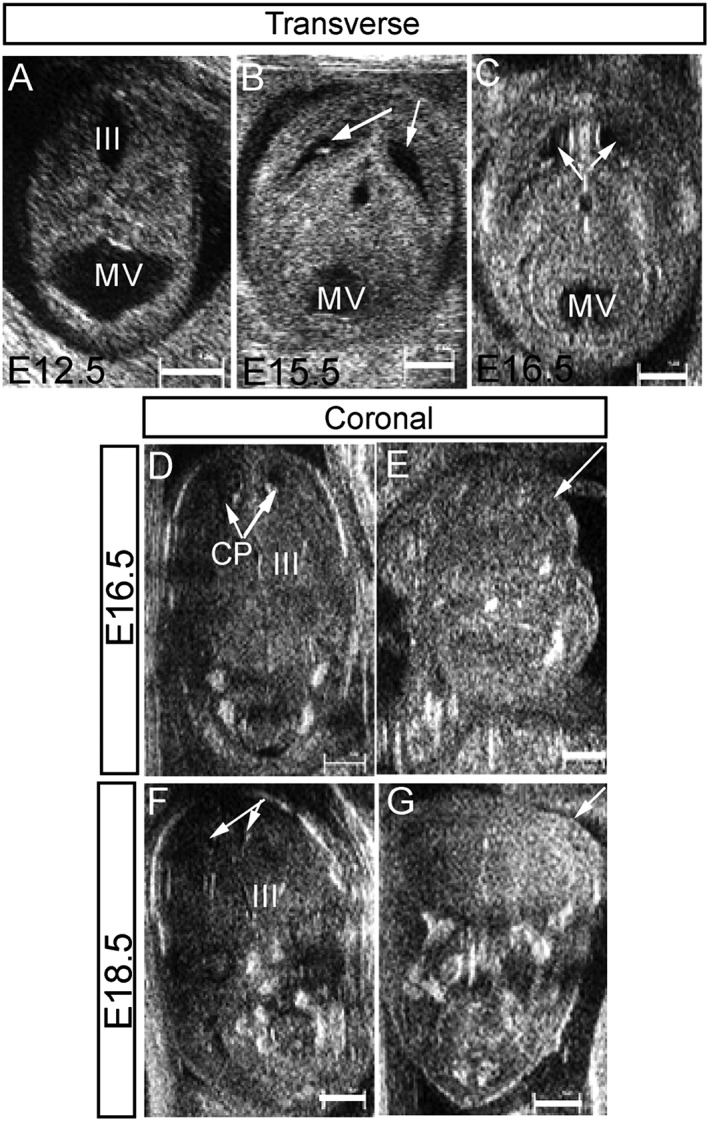
High‐frequency ultrasound imaging of wild‐type and *Gldc*‐deficient embryos: transverse sections of wild‐type embryos at E12.5 (A), E15.5 (B) and E16.5 (C) show the normal appearance of the mesencephalic vesicle (future aqueduct), the forming cerebral ventricles (arrows) and surrounding brain tissues. Coronal sections through the brain of a wild‐type embryo (D and F) analysed at both E16.5 (D) and E18.5 (F) illustrate the progressive reduction of volume of the body of the lateral ventricles with development. The choroid plexuses (CP) are detectable within the body of the lateral ventricles (arrows in D). The ultrasound appearance of exencephaly in *Gldc* ^*GT1/GT2*^ embryos was similar at E16.5 (E) and E18.5 (G); arrows indicate the protrusion of the brain tissues in E and G. (III, third ventricle; scale bar represents 1 mm)

A morphological examination of HFUS appearance of normal cerebral structures throughout embryonic development in *Gldc* mice was performed. At E12.5 and later stages, brain tissues were imaged as echogenic structures surrounding the cerebral ventricles, which appeared as anechoic fluid‐filled cavities. It was possible to distinguish the prominent lateral ventricles and the third ventricles arising from the forebrain vesicle anteriorly, and the mesencephalic vesicle (future cerebral aqueduct) arising from the hindbrain vesicle in the posterior part of the brain (Figure 3A–C). Starting from E16.5, it was possible to clearly discriminate both the body of the lateral ventricles and the base of the posterior horns of the lateral ventricles (Figures 2 and 3C). At these stages, the choroid plexuses were visible as echogenic structures arising from the medial wall of the lateral ventricles (Figure 3D) and from the roof and lateral walls of the fourth ventricle. It was not possible to discriminate between the plexus of the third ventricle, which arises from the roof of the pineal recess, and the surrounding brain tissue (Figure 3D and F).

At all gestational ages examined, midbrain–hindbrain exencephaly was detected by ultrasound as echogenic soft brain tissues extruding into the amniotic fluid (Figure 3E and G) and not covered by the cranial bones that are normally detectable (e.g. in Figure 3D and F). This finding was confirmed by post‐mortem examination of the embryos in all cases.

### Longitudinal examination of the cerebral ventricular system by HFUS

Longitudinal morphometric evaluation of the cerebral ventricular system (mesencephalic vesicle, body of the lateral ventricles, base of the posterior horn of the lateral ventricles and fourth ventricle), the cerebellum and the choroid plexus of the fourth ventricle was performed at E16.5 and E18.5.

Among wild‐type and heterozygous embryos, there was a significant decrease in the volume of the mesencephalic ventricles, body of the lateral ventricles and fourth ventricle between E16.5 and E18.5 (Table 3). At the same stages, there was a significant increase in the volume of the cerebellum at E18.5 compared with E16.5 (Table 3). These stage differences were apparent both in mean absolute volumes and in mean volumes normalised to hemisphere diameter.

**Table 3:** Volume of cerebral structures at E16.5 and E18.5 in wild‐type and *Gldc‐*deficient embryos

Cerebral structure	Embryonic day	Genotype/normalised volume (mm^3^ × 10^−2^)			
		*Gldc* ^+/+^	*Gldc* ^GT1/+^	*Gldc* ^GT2/+^	*Gldc* ^GT1/GT2^
Mesencephalic vesicle	16.5 18.5	30 ± 5 15 ± 2*	34 ± 4 13 ± 1*	35 ± 3 16 ± 2*	30 ± 3 14 ± 2*
Body of the LVs	16.5 18.5	15 ± 2 7 ± 1*	13 ± 2 8 ± 1**	11 ± 1 7 ± 1**	13 ± 3 23 ± 9***
Base of the posterior horn of LVs	16.5 18.5	10 ± 5 11 ± 5	10 ± 2 9 ± 1	13 ± 5 12 ± 3	13 ± 7 9 ± 6
Fourth ventricle	16.5 18.5	14 ± 2 7 ± 1*	12 ± 1 5 ± 1*	14 ± 2 4 ± 1*	10 ± 1 6 ± 1*
Fourth ventricle choroid plexus	16.5 18.5	95 ± 1 10 ± 1	10 ± 1 8 ± 1	11 ± 1 9 ± 4	80 ± 1 10 ± 1
Cerebellum	16.5 18.5	11 ± 0.01 22 ± 2*	13 ± 1 21 ± 3*	15 ± 1 19 ± 1*	12 ± 1 21 ± 2*

The mean normalised volume of the mesencephalic vesicle, body of the lateral ventricles (LVs) and fourth ventricle decreased from E16.5 to E18.5 in wild‐type and heterozygous embryos, while the volume of the cerebellum increased in all genotypes. The mean volume of the body of the LVs increased between E16.5 and E18.5 in homozygous *Gldc*‐deficient embryos. Number of embryos scanned, *n* = 13 *Gldc* ^+/+^, 13 *Gldc* ^GT1/+^, 4 *Gldc* ^GT2/+^ and 9 *Gldc* ^GT1/GT2^ at E16.5 and 11 *Gldc* ^+/+^, 9 *Gldc* ^GT1/+^, 4 *Gldc* ^GT2/+^ and 7 *Gldc* ^GT1/GT2^ at E18.5. Volumes are mm^3^ multiplied by 100.

* Significantly different to E16.5 of same genotype (*p* < 0.01).

** Significantly different to E16.5 of same genotype (*p* < 0.05).

*** Significant difference to wild type at same stage (*p* < 0.05).

**Figure 4:**
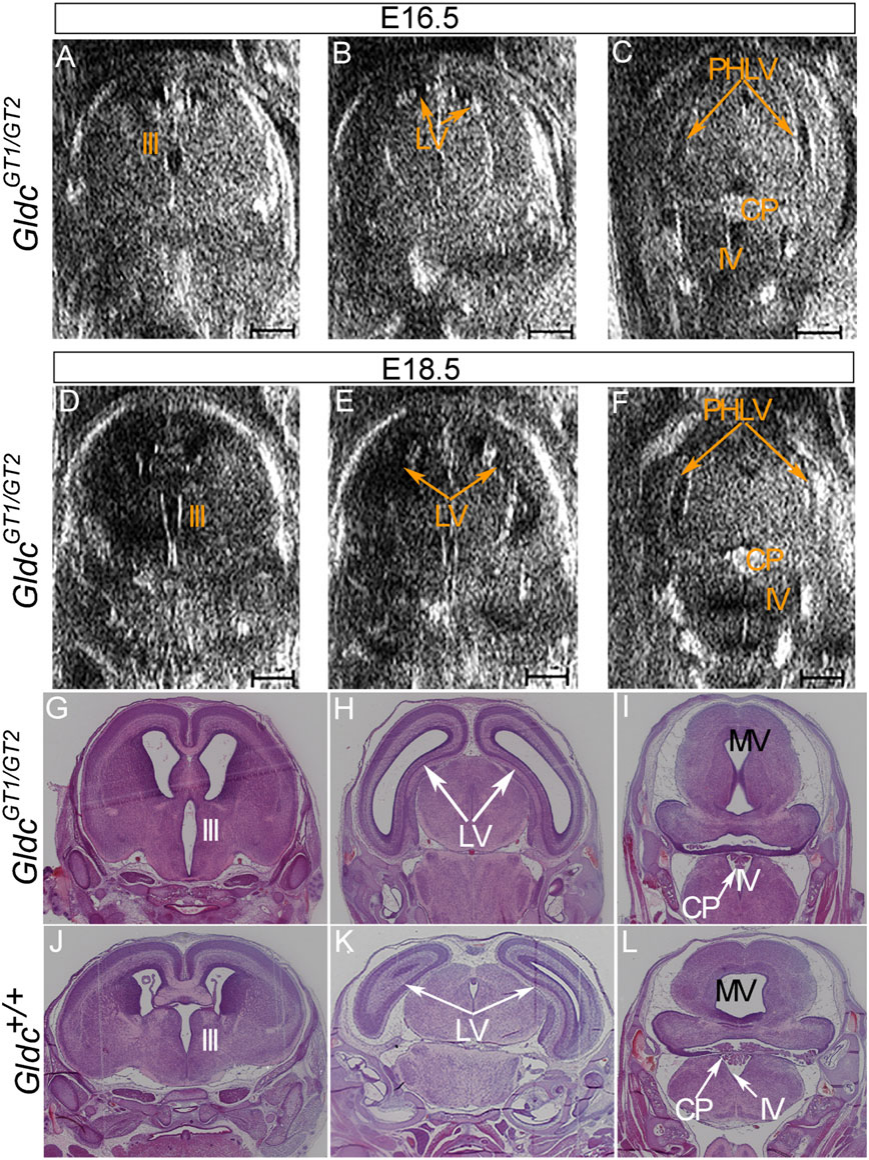
Development of ventriculomegaly observed by sequential high‐frequency ultrasound and histology: in a *Gldc* ^*GT1/GT2*^ embryo (A–F) high‐frequency ultrasound imaging of the brain revealed an enlargement of the body of the lateral ventricles at E18.5 (E) that was not evident at E16.5 (B). Mild enlargement of the third ventricle was also evident (A and D). The fourth ventricle did not appear to change in size from E16.5 to E18.5 (C and F). (Scale bar represents 1 mm). Histological coronal sections (G–L) in an anterior to posterior sequence (left to right) through the brain of *Gldc* ^*GT1/GT2*^ (G–I) and *Gldc* ^*+/+*^ (J–L) embryos at E18.5 confirm the abnormal appearance of the body of the lateral ventricles in the mutant (compare H and K), while the appearance of the fourth ventricle, including the choroid plexus, does not differ (compare I and L). MV, mesencephalic vesicle; LV, body of the lateral ventricles; PHLV, base of the posterior horn of the lateral ventricles; III, third ventricle; IV, fourth ventricle; CP, choroid plexus

Among the four genotypes, no significant differences were observed in the volume of the cerebral structures at E16.5 (Table 3), and mutant *Gldc* ^*GT1/GT2*^ embryos showed comparable volume changes at E16.5 to 18.5 in the mesencephalic vesicle, fourth ventricle and cerebellum as observed in the other genotypes (Table 3). In contrast, *Gldc*‐deficiency was associated with a significant increase in the volume of the body of the lateral ventricles as gestation advanced (instead of a reduction in volume as observed in wild types). As a result, the mean volume of the body of the lateral ventricles was significantly larger in *Gldc* ^*GT1/GT2*^ embryos at E18.5 than in the *Gldc* ^*+/+*^ group (Table 3). Analysis of the quantitative data as well as qualitative examination of 2D HFUS images of individual embryos at E16.5 and 18.5 showed that the difference in mean volume of the body of the lateral ventricles could be attributed to two of the seven *Gldc* ^*GT1/GT2*^ embryos (Figure 4E), accounting for the large intra‐group variability (Table 3).

### Confirmation of HFUS findings by histology

Within the group of *Gldc* ^*GT1/GT2*^ embryos analysed at E18.5, the two that exhibited enlargement of the body of the lateral ventricles on quantitative analysis also had an apparent enlargement of the third ventricle on 2D images (Figure 4D). In these two embryos, both the morphology and the volume assessment of the other brain regions did not show any obvious difference compared with wild‐type embryos (Table 3). Further detailed qualitative analysis of the cerebral ventricles at E16.5 showed that in one of the two embryos that later developed enlarged ventricles, a mild enlargement of the third ventricle was already visible (Figure 4A). The volume of this part of the ventricular system was not systematically measured because of its small dimension in the unaffected embryos.

The ultrasound findings were confirmed by histology at E18.5: a clear enlargement of the lateral and third ventricles structures above the fourth ventricle was evident in *Gldc* ^*GT1/GT2*^ embryos that showed HFUS abnormalities when compared with *Gldc* ^*+/+*^ littermates (Figure 4G–H compared with J and K). In contrast, *Gldc* ^*GT1/GT2*^ embryos with normal HFUS findings showed similar histology to *Gldc* ^*+/+*^.

## Discussion

Because of its non‐invasive nature, *in vivo* applicability to small animals and the high lateral and longitudinal spatial resolution with a tissue penetration of about 10 mm, HFUS has been recognised as an excellent imaging technology to follow *in utero* embryonic development in mice.^1^, ^17^, ^18^ In this study, we have used HFUS to characterise the phenotype of *Gldc‐*deficient mice, with subsequent confirmation of the HFUS findings by histological analysis. NTDs and hydrocephalus have both been associated with GCS mutation in humans,^7^, ^11^, ^13^ and we previously reported the corresponding presence of cranial NTDs and enlarged cerebral ventricles in *Gldc* mutant mice.^14^ However, the stage at which hydrocephalus develops in the mouse model had not been established, and this was complicated by the partial penetrance of this defect, which means that affected embryos could not be reliably identified soon after onset. The application of longitudinal HFUS, which allows retrospective analysis of those embryos that later exhibit a phenotype, highlights the potential value of this technique in understanding the pathogenesis of animal models for disease.

As shown at earlier stages,^19^ longitudinal evaluation during development was feasible by mapping each embryo, at different embryonic stages, relative to the position of the placenta within the amniotic sac and relative to the position of each sac in relation to maternal structures such as the bladder. In our study, we also used other maternal landmark structures such as the liver and spleen, and the embryo position within the uterine horn to allow us to follow the development of each embryo through serial ultrasound scans across gestation, and to recognise each embryo when dissected from the uterus at the end of gestation. We were able to correctly match ultrasound images with histological sections and genotypes. In this way, serial HFUS can be used to reduce the number of animals needed for phenotypic characterisation, in accordance with the philosophy of the 3Rs model of Russel and Burch (refinement, reduction and replacement). 3D reconstruction of embryo images also allows quantitative assessment of the structures of interest.

Mouse brain development has previously been studied from E10.5 to E14.5 using HFUS anular array.^1^ In that study, approximately 50% of each litter was imaged, using semi‐automatic segmentation of the whole ventricular system and subsequent normalisation of the ventricular volume to the volume of the whole head. In contrast in our study, we manually segmented the different parts of the ventricular system to better measure specific parts of the developing embryonic brain. We chose to obtain the 3D volumes with the skull in transverse section and in the midline so as to optimise reproducibility of the measurement, similar to the method used for clinical fetal imaging. The analysed volumes were then normalised to the ventricular hemisphere diameter rather than the total head volume to reduce the effect of non‐encephalic structures on the measurement. At E12.5, the lateral and third ventricles and the mesencephalic vesicle could all be identified. At later stages (E16.5 and E18.5), we could additionally identify the fourth ventricle with its choroid plexus and the cerebellum. Being fluid‐filled hypoechoic cavities, the cerebral ventricles and mesencephalic vesicle were all amenable to volume measurements at E16.5 and E18.5, with the exception of the third ventricle. In contrast, because of the similar echogenicity, it was much harder to distinguish the cerebellum volume from the adjacent structures, making this measurement in our opinion less accurate and reproducible.

At E12.5 and subsequent stages, irrespective of the position of the embryos and the probe used, we were able to identify exencephaly as a defect of the skull with exposure of brain tissue to the amniotic fluid. The exencephaly–anencephaly sequence is characterised by the progressive disruption of brain tissues exposed to the amniotic fluid with the production of a fibrotic and haemorrhagic mass with no functional cerebral cortex. In human embryos, this process is reflected by the change of the appearance of the brain on ultrasound throughout the pregnancy: in the first trimester, brain tissues that are not covered by the skull are seen as semicircular structures floating in the amniotic fluid in the coronal section (Mickey Mouse sign),^20^ while in the second trimester, no brain tissue is visible above the orbits plane (Frog sign). In the current study, the HFUS appearance of exencephaly in mouse embryos did not change from E15.5 to E18.5. A mass of heterogeneous density above the level of the orbits was still visible in images at E18.5 and confirmed on macroscopic examination.

The embryo position in the amniotic sac was one of the limiting factors in the acquisition of transverse and sagittal‐oriented volumes of the brain, especially at advanced embryonic stage (E18.5). Orientation of the ultrasound probe on the embryo was facilitated by extensive use of the tilt function of the imaging table and altering the angle of the probe. In this way, a suitable image of the brain necessary for qualitative and quantitative assessment of the structures of interest was obtained in the majority (88%) of the analysed embryos at this late gestation stage. When the desired plane was not achievable during the imaging acquisition, 3D volumes were acquired to provide a reconstruction of the images in the three planes, but with the consequent loss of details in the reconstructed plane. Moreover, the quality of HFUS images acquired at E18.5 was lower than at E16.5, due to the reduction of the amniotic fluid that provides natural contrast between the uterine wall and the mouse embryos. These technical difficulties account for the lower rate of success in the morphometric evaluation and the larger intra‐observer variability with the advancing gestation. An additional limitation of HFUS is the time of the acquisition of images for each litter, during which maternal circulation and body temperature must be monitored and may be influenced by the use of general anaesthesia.

Enlargement of the body of the lateral ventricles in affected *Gldc*‐deficient embryos was clearly visible in 2D HFUS images and was confirmed by volume assessment in the 3D reconstruction of the brain and by post‐mortem histological analysis at E18.5. One embryo, which subsequently developed enlarged ventricles, showed a mild enlargement of the third ventricle already present at E16.5 on qualitative examination of 2D images of the brain. This suggests that the onset of hydrocephalus in this mouse model is between E16.5 and E18.5, close to the end of the pregnancy. Strikingly, the HFUS appearance of the fourth ventricle and its choroid plexus was normal in both the mutant embryos with enlarged lateral and third ventricles, and these findings were confirmed on histological sections. Because only the structures above the aqueduct of Silvius were enlarged, we speculated that an obstruction at the level of the aqueduct might be the cause of hydrocephalus in this mouse model. At the same time, the analysis of 2D HFUS images of the base of the posterior horns of the lateral ventricles did not show any incontrovertible difference between wild‐type and mutant embryos. We hypothesise that the dilation of this part of the ventricular system might only be visible later in development or post‐natally compared with the enlargement of the body of the lateral ventricle. Further work will study in more detail the pathophysiology of this mouse mutant.

## Conclusion

This study confirms that both exencephaly and ventriculomegaly are detectable using HFUS during mouse embryonic development and indicates this technique to be an effective tool to study the development of the brain and the cerebral ventricles in the mouse embryo. Longitudinal ultrasound assessment is useful to evaluate changes of cerebral structures throughout fetal life and to detect alterations that occur at late gestational age.

**WHAT'S ALREADY KNOWN ABOUT THIS TOPIC?**

- High‐frequency ultrasound (HFUS) has been used to non‐invasively monitor *in utero* mouse central nervous system (CNS) development during mid‐gestation (embryonic day 10.5–14.5); later gestational ages are yet to be studied. Missense mutations of the glycine decarboxylase gene *(Gldc)* are associated clinically with a metabolic disorder, non‐ketotic hyperglycinemia (NKH) and neural tube defects (NTDs) such as exencephaly.

**WHAT DOES THIS STUDY ADD?**

- We extended HFUS imaging of the mouse CNS into late gestation embryonic day 18.5 in a genetic mouse mutant lacking *Gldc* in which NTDs, such as exencephaly, and hydrocephalus are prevalent. Serial HFUS can determine the age of onset of ventricle dilation that precedes hydrocephalus in this model.

## References

[1] Aristizabal O , Mamou J , Ketterling JA , Turnbull DH . High‐throughput, high‐frequency 3‐D ultrasound for in utero analysis of embryonic mouse brain development. Ultrasound Med Biol 2013;39:2321–32. 24035625 10.1016/j.ultrasmedbio.2013.06.015 PMC3834109

[2] Nieman BJ , Turnbull DH . Ultrasound and magnetic resonance microimaging of mouse development. Methods Enzymol 2010;476:379–400. 20691877 10.1016/S0076-6879(10)76021-3 PMC3160173

[3] Turnbull DH , Bloomfield TS , Baldwin HS , *et al.* Ultrasound backscatter microscope analysis of early mouse embryonic brain development. Proc Natl Acad Sci U S A 1995;92:2239–43. 7892254 10.1073/pnas.92.6.2239 PMC42459

[4] Turnbull DH . Ultrasound backscatter microscopy of mouse embryos. Methods Mol Biol 2000;135:235–43. 10791320 10.1385/1-59259-685-1:235

[5] Sudheendran N , Bake S , Miranda RC , Larin KV . Comparative assessments of the effects of alcohol exposure on fetal brain development using optical coherence tomography and ultrasound imaging. J Biomed Opt 2013;18:20506. 23386196 10.1117/1.JBO.18.2.020506 PMC3563965

[6] Go MK , Zhang WC , Lim B , Yew WS . Glycine decarboxylase is an unusual amino acid decarboxylase involved in tumorigenesis. Biochemistry 2014;53:947–56. 24467211 10.1021/bi4014227

[7] Narisawa A , Komatsuzaki S , Kikuchi A , *et al.* Mutations in genes encoding the glycine cleavage system predispose to neural tube defects in mice and humans. Hum Mol Genet 2012;21:1496–503. 22171071 10.1093/hmg/ddr585 PMC3298276

[8] Greene ND , Copp AJ . Neural tube defects. Annu Rev Neurosci 2014;37:221–42. 25032496 10.1146/annurev-neuro-062012-170354 PMC4486472

[9] Coughlin CR , Swanson MA , Kronquist K , *et al.* The genetic basis of classic nonketotic hyperglycinemia due to mutations in GLDC and AMT. Genet Med 2016 DOI: 10.1038/gim2016.74. 10.1038/gim.2016.74 27362913

[10] Hennermann JB , Berger JM , Grieben U , *et al.* Prediction of long‐term outcome in glycine encephalopathy: a clinical survey. J Inherit Metab Dis 2012;35:253–61. 22002442 10.1007/s10545-011-9398-1

[11] Hoover‐Fong JE , Shah S , Van Hove JL , *et al.* Natural history of nonketotic hyperglycinemia in 65 patients. Neurology 2004;63:1847–53. 15557500 10.1212/01.wnl.0000144270.83080.29

[12] Dobyns WB . Agenesis of the corpus callosum and gyral malformations are frequent manifestations of nonketotic hyperglycinemia. Neurology 1989;39:817–20. 2786166 10.1212/wnl.39.6.817

[13] Van Hove JL , Kishnani PS , Demaerel P , *et al.* Acute hydrocephalus in nonketotic hyperglycemia. Neurology 2000;54:754–6. 10680820 10.1212/wnl.54.3.754

[14] Pai YJ , Leung KY , Savery D , *et al.* Glycine decarboxylase deficiency causes neural tube defects and features of non‐ketotic hyperglycinemia in mice. Nat Commun 2015;6:6388. 25736695 10.1038/ncomms7388 PMC4366506

[15] Kaufman MH . The Atlas of Mouse Development. Academic Press: London; 1992.

[16] Heijman E , Aben JP , Penners C , *et al.* Evaluation of manual and automatic segmentation of the mouse heart from CINE MR images. J Magn Reson Imaging 2008;27:86–93. 18050352 10.1002/jmri.21236

[17] Norris FC , Wong MD , Greene ND , *et al.* A coming of age: advanced imaging technologies for characterising the developing mouse. Trends Genet 2013;29:700–11. 24035368 10.1016/j.tig.2013.08.004

[18] Phoon CK . Imaging tools for the developmental biologist: ultrasound biomicroscopy of mouse embryonic development. Pediatr Res 2006;60:14–21. 16690959 10.1203/01.pdr.0000219441.28206.79

[19] Ji RP , Phoon CK . Noninvasive localization of nuclear factor of activated T cells c1−/− mouse embryos by ultrasound biomicroscopy‐Doppler allows genotype–phenotype correlation. J Am Soc Echocardiogr 2005;18:1415–21. 16376776 10.1016/j.echo.2005.04.006

[20] Chatzipapas IK , Whitlow BJ , Economides DL . The ‘Mickey Mouse’ sign and the diagnosis of anencephaly in early pregnancy. Ultrasound Obstet Gynecol 1999;13:196–9. 10204212 10.1046/j.1469-0705.1999.13030196.x

